# Development and psychometric properties of the maternal ambivalence scale in spanish women

**DOI:** 10.1186/s12884-022-04956-w

**Published:** 2022-08-06

**Authors:** Magdalena Belén Martín-Sánchez, Verónica Martínez-Borba, Patricia Catalá, Jorge Osma, Cecilia Peñacoba-Puente, Carlos Suso-Ribera

**Affiliations:** 1 Department of Basic and Clinical Psychology and Psychobiology, Jaume I University, Castellón, Spain; 2 Clínica Gaitán. El Puerto de Santa María, Cadiz, Spain; 3 Department of Psychology, Rey Juan Carlos University, Alcorcón, Madrid, Spain; 4 Departament of Psychology and Sociology, Universidad de Zaragoza, Teruel, Spain; 5 Instituto de Investigación Sanitaria de Aragón, Zaragoza, Spain; 6 CIBER of Physiopathology of Obesity and Nutrition CIBERobn, Instituto de Salud Carlos III, Madrid, CB06/03 Spain

**Keywords:** Maternal ambivalence; pregnancy, Postpartum, Questionnaire development psychometric properties

## Abstract

**Background:**

maternal ambivalence, which refers to experiencing mixed emotions about motherhood, like happiness and sadness, is frequent during the perinatal period.

**Aim:**

Due to the relevance of this topic and the lack of psychometrically-sound instruments to measure it, this study aims to develop and test a measure of maternal ambivalence called the Maternal Ambivalence Scale (MAS).

**Methods:**

in this cross-sectional, observational study, participants were 1424 Spanish women recruited online who were either pregnant (33%) or recent mothers of children under 2 years (67%). They responded to the MAS and measures of anxiety and depressive symptoms and life satisfaction. Analyses included exploratory and confirmatory factor solutions for the MAS, internal consistency estimates (Cronbach’s α) for all scales, as well as bivariate correlations to investigate sources of validity evidence. Comparisons between pregnant and postpartum women were also examined.

**Results:**

The assumptions for factor analysis about the relationship between items were met (Kaiser-Meyer-Olkin’s [KMO] test = 0.90; Barlett’s Chi-square sphericity test = 5853.89, *p* < .001). A three-factor solution (Doubts, Rejection, and Suppression) for the MAS showed a good model fit both in exploratory (Chi-square = 274.6, *p* < .001, Root Mean Square Error of Approximation [RMSEA] = 0.059, RMSEA 90% Confidence Interval [CI]=[0.052, 0.066], Comparative Fit Index [CFI] = 0.985, Tucker Lewis Index [TLI] = 0.974) and confirmatory analyses (Chi-square = 428.0, *p* < .001, RMSEA = 0.062, RMSEA 90% CI=[0.056, 0.068], CFI = 0.977, TLI = 0.971). Doubts (α = 0.83), Rejection (α = 0.70), and Suppression (α = 80) were associated with higher anxiety and depressive symptoms, as well as lower life satisfaction (all *p* < .001). Pregnant women presented greater Rejection (mean difference = 0.30, *p* = .037, 95% CI=[0.02, 0.58]) and less Suppression (mean difference=-0.47, *p* = .002, 95% CI=[-0.77,-0.17]) than mothers.

**Conclusion:**

with this study, we provide clinicians and researchers with a novel tool that successfully captures the complex nature of maternal ambivalence. Given the associations of maternal ambivalence with important outcomes in perinatal women, this tool could be important for the prevention of distress associated with chronic ambivalence and to evaluate the effectiveness of interventions addressing ambivalence.

**Supplementary Information:**

The online version contains supplementary material available at 10.1186/s12884-022-04956-w.

## Background

A traditional belief, which is still present in some societies and individuals today, is that every woman should be willing to become a mother and crave motherhood. Under this assumption, becoming a mother should be associated with feelings of happiness and joy [1]. Research, however, has also indicated that motherhood can be associated with negative experiences, including loss in self-esteem and self-confidence due the responsibilities imposed by this new role, body image dissatisfaction, difficulties in maintaining important areas for their quality of life, such as social relationships, work, and independence/leisure, and mental distress, in the form of anhedonia, resentment, boredom, stress, depression, and anxiety [2, 3].

This coexistence of both positive and negative feelings associated with being a mother had led to the popularization of the term “maternal ambivalence”. Ambivalence refers to the presence of simultaneous conflictive reactions towards the same object, person or action curse, which is manifested at a cognitive, an affective, and/or a behavioural level [4, 5]. While several definitions of ambivalence exist [6–9], two elements are key: (i) the presence of positive and negative associations about the same attitude and (ii) the same level of relevance in both associations, that is in positive and negative ones [10]. In particular, maternal ambivalence is used to describe mixed, simultaneous positive and negative feelings, thoughts, and behaviours from the mother towards being a mother (e.g., newly imposed roles) or towards the baby and can affect all women, both actual or potential mothers [11].

The term “maternal ambivalence” was proposed over a hundred years ago by Freud and then further developed by Klein and Winnicott in the mid-fifties, but its popularity in psychology research has only boosted in the last decades [4]. While ambivalence has been argued to be an inherent, non-pathological process of motherhood [12], research has also shown that, if inadequately addressed (e.g., if ignored or even suppressed), the conflict that arises from ambivalence may cause important consequences on the mental health of women [13, 14]. Thus, it is important to organize resources to provide assistance and follow-up to women who experience a significant degree of interference due to maternal ambivalence [2].

A problem with research into maternity, however, lies in the absence of unanimity in the conceptualization and operationalization of maternal ambivalence [15]. Particularly, a conceptually robust and psychometrically-sound measure of maternal ambivalence is missing. For example, this construct has been often evaluated using ad hoc single item questions (e.g., “to what extent have you experienced mixed positive and negative feelings about maternity”) without a proper scale development procedure [16, 17] or using qualitative interviews [2]. Other authors have used indirect indicators, such as adherence to oral contraceptive treatment, as indicators of maternal ambivalence [18, 19], which is again likely to be a too reductionist and inaccurate representation of a complex construct like maternal ambivalence. This study aimed to develop a theoretically and psychometrically strong measure of maternal ambivalence.

So far, studies reveal three common elements that seem to be involved in the experience of maternal ambivalence [20]. The first refers to the presence of *doubts* about becoming a mother, about being a good mother, or about one’s own will to want to be a mother [2, 12, 21]. Doubts appear in the form of thoughts and feelings of regret or an urge to “back off” or disappear, especially in the weeks just before and immediately after birth [2], and have been linked to feelings of insecurity or inadequacy, fear of rejection, and fear about the transition to motherhood [2, 12, 14]. The second common element in research into maternal ambivalence is the degree of *conviction* about becoming a mother [2, 12, 21, 22]. This conviction or confidence about becoming a mother is defined differently across investigations. Some studies focus on the positive meaning attributed to motherhood and the will to be mothers [2, 22]. Other times, conviction is evaluated by means of the losses associated with becoming a mother that affect certainty about being a mother or magnify the lack of it [2, 12, 21]. The third and final component of maternal ambivalence that emerges from the literature is the *coping* strategy implemented to deal with ambivalence. Coping can be understood as the cognitive and behavioural efforts aimed to solve specific situations that are perceived as threatening and demanding [23]. In the context of motherhood, coping has been conceptualized in a continuum between approach and avoidance. Approach coping strategies are understood as the search for support and information in planning and preparing for motherhood, while avoidance would be represented by efforts to minimize the confrontation of tasks associated with preparing for maternity [14, 24].

Because a psychometrically and conceptually sound measure that captures the complex nature of maternal ambivalence is missing, this study aims to develop and test a measure of maternal ambivalence, namely the Maternal Ambivalence Scale (MAS). The MAS includes items that evaluate each of three elements of maternal ambivalence described above, that is, doubts, conviction, and coping. Our goal is therefore to create a set of representative items of these three components of maternal ambivalence based on past research and evaluate the psychometric properties of the MAS in terms of factor structure, internal consistency, and sources of construct validity evidence. To do so, the scale has been administered both to pregnant women and to women who have recently given to birth (< two years since delivery) because pregnant women have been argued to experience ambivalence as more unpleasant because they cannot make a number of decisions about pregnancy (i.e., accelerate the gestation period and, depending on the trimester, they cannot interrupt the pregnancy either) and also do not yet experience the benefits of interacting with the baby [14]. In addition to testing the three-factor internal structure of the scale (doubts, conviction/rejection, and coping/suppression), which we expect to confirm, we investigate sources of construct validity evidence of the MAS in relation to measures of depression, anxiety, and satisfaction with life. In line with past research [14, 17], we expect that maternal ambivalence will be associated with more emotional suffering (i.e., depression and anxiety) and reduced life satisfaction.

## Participants, ethics and methods

### Procedure and sample

This is a cross-sectional, observational study conducted completely online. The Ethics Committee of the Jaume I University reviewed the present study protocol and management of confidentiality and personal data and approved all the present study procedures. These consisted of the elaboration of an online assessment protocol, which was uploaded into the Qualtrics online survey platform in a format that preserved the anonymity of the participants. Subsequently, an online link to the survey was massively disseminated using paid advertisement on social networks (i.e., Facebook), as well as associations of midwives and online groups aimed to support during preparation for childbirth from different autonomous communities in Spain. The instruments included in this survey will be described in the Measures section. The entire survey took approximately 10 min to complete. Before completing the measures, the potential participants were asked about their eligibility and their willingness to participate. This was also conducted online within the same protocol, after showing the study information sheet. If they provided their consent to participate in the online Qualtrics platform and met the eligibility criteria, they were allowed to complete the assessment protocol.

The sample to validate this scale consisted of women of legal age (≥ 18 years) who were pregnant or were mothers of at least one child between 0 and 2 years (i.e., inclusion criteria). This age period was chosen since the literature indicates that this is the postpartum stage in which postpartum anxiety/depressive symptoms are more frequent and intense [25]. Other inclusion criteria included understanding Spanish and being born or currently living in Spain. Participants were also required to have a device with Internet access since the study dissemination was made using social networks and the assessment protocol was completely online. Women were excluded if they were not pregnant or did not have a baby in the past two years and if they were not born in Spain or currently living in Spain.

In total, 1774 women accessed the survey from April to June 2021. Of these, data from 1424 were valid (*n* = 14 did not report their age, *n* = 266 indicated that they were not pregnant or did not have a baby of between 0 and 2 years, and *n* = 70 were not Spanish and were not living in Spain). The sample data was collected from April 6 to April 28 of 2021. The required sample size was calculated based on expert recommendations that suggest a minimum of 300 to 450 to obtain sufficiently comparable patterns in scale development [26], over 1,000 participants for excellent estimates in this type of studies [27], and at least 10 participants for item [28].

### Measures

#### Development of the maternal ambivalence scale (MAS)

Because multi-item scales as opposed to single item scales are generally recommended [29] and at least three items for each construct are required to obtain satisfactory internal consistency estimates [30, 31], a minimum of 4 items were created in the first stage of scale development. This was done to ensure that, even if some items presented poor psychometric properties, the final subset of items after removing problematic ones would be sufficient for each factor. Finally, 14 items were created to evaluate the three components of maternal ambivalence: doubts (*n* = 6), conviction/rejection of motherhood (*n* = 4), and coping/suppression (*n* = 4).

#### Translation and adaptation of the items in the MAS

As noted earlier, items on the MAS were developed following previous literature on the field and included three elements of maternal ambivalence, namely Doubts, Conviction (which we have labelled Rejection of motherhood), and coping (which we have labelled Suppression). Scale development was particularly inspired by the Stafford clinical interview [32] and the semi-structured interview by Cutler et al. [16]. For example, item 10 (“When I think about motherhood, I have positive and negative feelings”) from the doubts scale was created after reading items in the Stafford Interview [32] that evaluated adaptation to pregnancy (i.e., very positive to very negative reaction). Conviction/Rejection, as in item 4 (“Being a mother means advancing and evolving in my life”), was inspired by questions such developed by Cutler et al. [16] such as the degree of sacrifice the mother was making, which ranges from no sacrifice to great sacrifices. In this case, the item was reformulated to evaluate the conviction of being a mother as a compensatory element of the costs experienced. To evaluate the presence of negative emotions associated with motherhood, such as fear or directly rejection (item 7 of the MAS: “Sometimes I am assaulted by a great rejection or fear regarding motherhood”), the question “What were the main concerns and / or fears during this pregnancy?” from the Cutler et al. [16] interview was taken as reference. Finally, coping included efforts to suppress and express ambivalence and perceived support [33, 34]. The main support figures were selected from the Stafford Interview [32]. For example, item 11 refers to the family (“If I had doubts about motherhood, I would share them openly with my family”), while items 12 and 13 refer to friends and the romantic partner, respectively. Item 14 collects information on whether the woman tends to suppress as a coping strategy (“If I had doubts about motherhood, I would surely keep them to myself”). In general, items were translated or adapted by two bilingual (Spanish-English) members of our team with expertise in this type of procedure and then agreement was reached with the rest of the members of the group.

Then, these items were presented to a committee of 5 experts, who made some writing suggestions and confirmed the suitability of the items. The 5 experts included two clinical psychologists with over eight years of experience in perinatal mental health care, including research, one nurse with six years of clinical experience in perinatal care, an experienced midwife, and a gynaecologist with over ten years of clinical experience. The content validity of the items as appraised by this panel of experts was also quantitatively assessed with the Content Validity Index and the Content Validity Ratio, which will be described in the Data analysis section.

#### Final version of the MAS

The final version of the MAS can be found in Appendix I. The answers in the MAS are responded using a Likert scale ranging from 1 (Completely disagree) to 4 (Completely agree). The degree of agreement was distributed in 4 response options to avoid central tendency bias. To facilitate the calculation of a total ambivalence score, positively worded items reflecting conviction/rejection (items 1, 4, 6, and 9) and coping/suppression (items 12, 13, and 14) were recoded before the factor analysis so that high scores in each factor would reflect high ambivalence (i.e., frequent doubts, rejection of motherhood, and a tendency to suppress as opposed to express ambivalence). For clarity reasons, from now on the MAS scales will be named: Doubts, Rejection, and Suppression. The English and Spanish versions of the scale can be found as Supplementary material.

#### Sources of content validity evidence

At the end of the item development process, the set of 14 items of the MAS were shown to the five experts together with a rating scale to evaluate the degree of relevance of each item. As recommended in past research [35], a Likert scale with four response options was used, where “1 = the item is not relevant for the assessment of maternal ambivalence”, “2 = the item is somewhat relevant for the assessment of maternal ambivalence”, “3 = the item is quite relevant for the assessment of maternal ambivalence”, and “4 = the item is highly relevant for the assessment of maternal ambivalence”.

#### Sources of construct validity evidence

In addition to MAS, the assessment protocol included a series of measures to evaluate sources of construct validity evidence of the MAS. These were two screening instruments for anxiety and depression, that is, the Overall Anxiety Severity and Impairment Scale (OASIS) [36, 37] and the Overall Depression Severity and Impairment Scale (ODSIS) [36, 38], and a measure of satisfaction with life, namely the Life Satisfaction Scale (SWLS) [39, 40]. The OASIS and the ODSIS identify the frequency, intensity and interference or deterioration caused by the symptoms on the personal and social levels using 5 items each. Responses are ranked using a Likert-type scale from 0 to 4, where 0 represents “not at all anxious/depressed” and 4 means “constantly anxious/depressed”. Total scores in the OASIS and ODSIS can range from 0 to 20 and higher scores reflect greater deterioration of daily living due to anxiety or depressive symptoms. The SWLS measures the perception of well-being that a person experiences using 5 items. The responses are distributed on a Likert-type scale in a range from 1 (Strongly disagree) to 7 (Strongly agree), so total scores have a 5–35 range where higher scores reflect greater life satisfaction. The internal consistency estimates (Cronbach’s alphas) of the OASIS, the ODSIS, and the SWLS in the present sample were 0.91, 0.94, and 0.85, respectively.

### Data analysis

We first analysed the sociodemographic data of the sample using descriptive statistics (means, standard deviations, and frequencies) to characterize the population. Next, we focused on the content validity of the items, as appraised by the committee of five experts. As recommended in past research [41], the Content Validity Index (CVI), which is the most frequently used measure of construct validity, was computed. The CVI is calculated as the number of items in which the experts indicated a “3 = the item is quite relevant for the assessment of maternal ambivalence” or a “4 = the item is highly relevant for the assessment of maternal ambivalence”, divided by the number of items. With five experts, CVI values, which range from 0 to 1, should achieve the maximum of 1 [42].

The following step was to evaluate the characteristics of the items in the MAS, including means, standard deviations, kurtosis, and skewness. Recommended values for skewness and kurtosis are within a -2/+2 range [43]. Next, we explored whether the expected three-factor structure of the MAS obtained sufficiently good evidence of fit to the data. For this purpose, we conducted both an Exploratory and a Confirmatory Factor Analysis with MPlus version 6.12 [44] using the following fit indices: the comparative fit index of the scale (CFI), the Tucker-Lewis index (TLI), Chi square (χ2), and the root mean square error of approximation (RMSEA). CFI and TLI values above 0.95 are often interpreted as revealing an excellent fit. It is argued that RMSEA values less than 0.05 show an excellent fit of the data model to the model, while scores less than 0.08 are interpreted as indicative of a good fit [45, 46]. The combination of exploratory and confirmatory analyses would allow to investigate whether the proposed three-factor solution had good fit to the data (confirmatory analysis) and whether the solution proposed was the optimal for the data or alternative solutions were preferable (exploratory analysis). The cut-off used for factor loadings was 0.4 [47]. Internal consistency estimates (Cronbach’s alphas) are also reported.

As a final step, we computed a series of Pearson bivariate associations between the MAS and the measures of construct validity evidence (OASIS, ODSIS, and SWLS), as well as Student’s t tests to compare scores in the MAS in the two groups of participants: pregnant women and mothers with children aged 0 to 2 years. The descriptive analyses, the Pearson correlations, and independent samples t-tests were computed with IBM SPSS Statistics version 26.0 [48]. Normality and homogeneity of variances assumptions will also be investigated by means of the Kolmogorov-Smirnov and the Levene’s test.

## Results

### Sample characteristics

In total, 1424 women participated into the study (mean age = 34.7 years; *SD* = 4.9 years; range between 18 and 50 years). Of these, 33.0% were pregnant (mean age = 33.7 years; *SD* = 4.8 years; range between 21 and 47 years) and 67.0% were mothers with children between 0 and 2 years (mean age = 35.3 years; *SD* = 4.8 years; range between 18 and 50 years). Most pregnant women were in the third trimester (41.0%), followed by women in the second (33.8%), and finally first trimester (25.2%). From the eligible participants, 87.6% were born in Spain and 93.2% were living in Spain.

Regarding marital status, the majority of the participants were in a relationship at the time of assessment (90.0%). Most of them reported being heterosexual (94.5%). Bisexual (3.6%), homosexual (0.9%), and other sexual orientations (1.0%) were less frequent.

With respect to the educational level, most women had university or higher studies (70.5%). A smaller percentage of participants had completed technical studies (19.6%), while only 9.9% of the sample had finished secondary or primary studies.

Half of the participants were actively working at the time of assessment (54.4%). A significant number of women, however, were either on sick leave (20.7%), unemployed (18.8%), or housewives (6.1%).

Finally, when the participants were asked whether they had been diagnosed with a psychiatric disorder in the last 5 years, 2.6% indicated that they had received a diagnosis of major depressive disorder, 1.8% of post-traumatic stress disorder, 1.4% of panic disorder, 1.3% of obsessive-compulsive disorder, 8.2% of generalized anxiety disorder, 0.7% of dysthymic disorder, 0.6% of specific phobia, 0.4% of agoraphobia, 0.3% of social anxiety disorder, and 0.2% of bipolar disorder. In total, 14.5% of women indicated that they were receiving mental health treatment at the time of the assessment. In particular, 10.5% were receiving psychotherapy, 2.2% reported receiving psychiatric treatment, and 1.8% informed that they were receiving both forms of therapy.

### Content validity of the items

The results of the content validity evaluation revealed a perfect CVI score, because all the items were given a score of “3 = the item is quite relevant for the assessment of maternal ambivalence” or “4 = the item is highly relevant for the assessment of maternal ambivalence” by all the experts. Specifically, of the 70 evaluations (14 items * 5 panellists), the maximum appraisal (“4”) was received 65 times and a “3” was obtained 5 times. None of the items received more than one “non-excellent” evaluation, so all the items were retained.

### Descriptive analysis of the items in the MAS

Items in the MAS, which ranged from 1 (Completely disagree) to 4 (Completely agree), had a mean of between 1.56 and 2.84 and a standard deviation of between 0.73 and 1.01 in the population. No kurtosis and skewness problems were observed, as skewness values for all items ranged from − 0.59 to 1.12 and kurtosis ranged from − 1.05 to 1.21.

### Exploratory and confirmatory factor analyses

The results of the Kaiser-Meyer-Olkin’s (KMO) measure of sampling adequacy (KMO = 0.90) and the Barlett’s sphericity test (Chi-square = 5853.89, p < .001) supported the assumptions about the strength of the partial correlation between the MAS items and the non-identity of the correlation matrix (sufficient relationship between items), so the items were adequate for factor analysis. The exploratory factor analysis was conducted for models with up to four factors for parsimony reasons. As reported in Table 1, fit indices for one- and two-factor models did not support the fit of these models to the data. Exploratory models with three (Chi-square = 274.6, *p* < .001, RMSEA = 0.059, RMSEA 90% CI = [0.052, 0.066], CFI = 0.985, TLI = 0.974) and four factors (Chi-square = 163.1, *p* < .001, RMSEA = 0.049, RMSEA 90% CI = [0.041, 0.057], CFI = 0.992, TLI = 0.982) were the first to obtain adequate evidence of fit. However, the three-factor solution was preferred for parsimony, because in the four-factor solution item loadings in factor 4 were generally low (all below 0.35), and the item with the highest loading on this factor had a higher loading on another factor (factor 2; loading = 0.45). The three-factor solution was also preferred because it was conceptually consisted with the theory used to develop the scale. In the three factor solution, each factor included at least 4 items, as suggested by guidelines [29]. The three-factor solution and item distribution proposed by the exploratory factor analysis was consistent with the solution we anticipated and implemented in the confirmatory analyses, which also showed a good fit (Chi-square = 428.0, p < .001, RMSEA = 0.062, RMSEA 90% CI = [0.056, 0.068], CFI = 0.977, TLI = 0.971). Specifically, item distribution was as follows: items 2, 3, 5, 7, 9, and 10 loaded onto a factor which we originally labelled as “Doubts”, items 1, 4, 6, and 8 loaded onto a “Rejection” factor, and items 11 to 14 loaded onto the “Suppression” factor. As indicated in Table 2, which reflects the factor loadings of the items as indicated in the confirmatory factor analysis, the factor loadings of the items were all satisfactory.

**Table 1:** Fit indices of the exploratory and confirmatory analyses

Model	Chi square	p	RMSEA	RMSEA 90% CI	CFI	TLI
*Exploratory*						
1 factor	2898.5	< 0.001	0.172	0.166, 0.177	0.815	0.781
2 factors	738.5	< 0.001	0.092	0.086, 0.098	0.956	0.937
3 factors	274.6	< 0.001	0.059	0.052, 0.066	0.985	0.974
4 factors	163.1	< 0.001	0.049	0.041, 0.057	0.992	0.982
*Confirmatory*						
3 factors	428.0	< 0.001	0.062	0.056, 0.068	0.977	0.971

**Table 2:** Factor loadings and item distribution in the Maternal Ambivalence Scale according to the three-factor solution tested in by confirmatory factor analyses

	Doubts						Rejection				Suppression			
Item	2	3	5	7	9	10	1	4	6	8	11	12	13	14
Loading	0.83	0.66	0.87	0.76	0.79	0.52	0.71	0.69	0.92	0.52	0.76	0.68	0.84	0.84

All loadings *p* < .001

The Confirmatory Factor Analysis (CFA) confirmed the good fit of the three-factor model. In this three-factor solution, we also allowed the three factors to load onto a second-order factor, which we named “Maternal ambivalence total score”. The factor loadings were all above the recommended cut-off of 0.32 [49]: doubts = 0.80, lack of conviction = 0.62, and suppression = 0.37. All items showed significant, moderate-lo-large factor loadings (Table 2).

The analysis of internal consistency also showed good indicators for the three dimensions. Specifically, the Cronbach’s alpha of the Doubts, Rejection, and Suppression dimensions were 0.83, 0.70, and 0.80, respectively (Table 3). Again, to evaluate whether a total ambivalence score could also be calculated, an internal consistency estimate was calculated using all items in the scale. The Cronbach’s alpha of the MAS total score was 0.86.

**Table 3:** Bivariate Pearson correlations between the three dimensions of ambivalence (doubts, conviction, and coping) and anxiety, depression, and life satisfaction, together with means and standard deviations and internal consistency estimates (Cronbach’s alphas)

	Mean (SD)	Cronbach’s α	2	3	4	5	6	7
1. Doubts	11.69 (3.55)	0.83	. 58*	0.36*	0.88*	0.42*	0.46*	− 0.38*
2. Rejection	6.95 (2.36)	0.70		0.30*	0.78*	0.23*	0.28*	− 0.30*
3. Suppression	7.87 (2.55)	0.80			0.68*	0.26*	0.30*	− 0.37*
4. Total MAS	26.48 (6.71)	0.86				0.40*	0.45*	− 0.45*
4. Anxiety	9.05 (4.02)	0.91					0.73*	− 0.39*
5. Depression	8.46 (4.17)	0.94						− 0.46*
6. Life satisfaction	23.84 (6.35)	0.85						

*p < .001; *MAS *Maternal Ambivalence Scale

### Sources of construct validity evidence

Finally, we conducted a series of Pearson correlations between the MAS and the OASIS, the ODSIS, and the SWLS to check evidence of construct validity of the newly developed ambivalence scale. These are shown in Table 3.

The first factor (Doubts), correlated moderately with the other two dimensions of the same scale, namely Rejection (*r* = .58; *p* < .001) and Suppression (*r* = .36; *p* < .001). Regarding the measures of anxiety and depression, the Doubts scale showed moderate positive correlations both with the OASIS (*r* = .42; *p* < .001) and the ODSIS (*r* = .46; *p* < .001). The correlation of this first dimension of maternal ambivalence with SWLS was negative and presented a moderate strength (*r* = − .38; *p* < .001).

The second factor (Rejection) had positive and moderate associations with Suppression (*r* = .30; *p* < .001). Additionally, Rejection presented positive, modest correlations with the OASIS (*r* = .23; *p* < .001) and the ODSIS (*r* = .28; *p* < .001), as well as modest and negative associations with life satisfaction (*r* = − .30; *p* < .001).

Finally, the third factor (Expression) showed positive associations with the OASIS (*r* = .26; *p* < .001) and the ODSIS (*r* = .30; *p* < .001) and negative correlations with the SWLS scale (*r* = − .37; *p* < .001). The total MAS score correlated significantly and moderately with the OASIS (*r* = .40; *p* < .001), the ODSIS (*r* = .45; *p* < .001), and life satisfaction (*r* = − .45; *p* < .001). Finally, the OASIS and the ODSIS were strongly associated (*r* = .73; *p* < .001), while the correlation between SWLS and the OASIS (*r* = − .39 *p* < .001) and the ODSIS (*r* = − .46; *p* < .001) were moderate and negative.

### Differences in maternal ambivalence, anxiety, depression, and satisfaction with life between pregnant women and mothers of children between 0 and 2 years

Normality and homogeneity of variances assumptions were both met for the MAS scales and the total MAS score, as indicated by the Kolmogorov-Smirnov test (all *p* < .05) and the Levene’s test (all p > .05). The analyses of differences between pregnant women and mothers of children between 0 and 2 years, which are reported in Table 4, revealed significant differences in two dimensions of ambivalence, namely Rejection (pregnancy: mean = 7.15, SD = 2.34; mothers: mean = 6.85, SD = 2.36; mean difference = 0.30, *p* = .037, 95% CI = [0.02,0.58]) and Suppression (pregnancy: mean = 7.56, SD = 2.47; mothers: mean = 8.03, SD = 2.57; mean difference = -0.47, *p* = .002, 95% CI = [-0.77, -0.17]). Pregnant women were less likely to feel convinced about being mothers when compared to actual mothers and mothers were less likely to express their ambivalence to others than pregnant women. No differences were found between the two samples in doubts or in anxiety, depression, and satisfaction with life.

**Table 4:** Independent sample’s Student t-test comparing pregnant women and mothers of children between 0 and 2 years in the study variables

	Pregnant Mean (SD)	Mother Mean (SD)	Mean difference	p	95% CI	Cohen’s d
Doubts	11.95 (3.54)	11.56 (3.55)	0.39	0.072	-0.03, 0.81	0.11
Rejection	7.15 (2.34)	6.85 (2.36)	0.30	0.037	0.02, 0.58	0.12
Suppression	7.56 (2.47)	8.03 (2.57)	-0.47	0.002	-0.77, -0.17	0.19
MAS total	26.61 (6.63)	26.42 (6.76)	0.18	0.653	-0.61, 0.98	0.03
Anxiety	8.84 (3.95)	9.14 (4.05)	-0.30	0.250	-0.81, 0.21	0.07
Depression	8.48 (4.24)	8.44 (4.14)	0.04	0.893	-0.49, 0.56	0.01
Life satisfaction	24.36 (6.16)	23.59 (6.42)	0.77	0.051	-0.01, 1.54	0.12

*MAS* Maternal Ambivalence Scale

## Discussion

The present study aimed at developing a new measure of maternal ambivalence that could be easily administered to women. In doing so, we also aimed test whether the scale presented good evidence in terms of internal consistency (consistency with the theoretical three-factor structure) and validity (correlations with measures of anxiety, depression, and satisfaction with life). In sum, the analyses supported the theorized three-factor solution, which we labelled as Doubts, Rejection, and Suppression, and these dimensions of ambivalence correlated with measures of anxiety, depression, and life satisfaction in the expected direction. Finally, we found differences in maternal ambivalence according to the perinatal moment, with pregnant women presenting more Doubts and showing a more expressive as opposed to suppressive ambivalence coping style than recent mothers. Overall, these findings suggest that the development of the scale was successful and indicate that this might be a clinically relevant tool to be used in women at different perinatal stages.

Maternal ambivalence is a complex construct. According to past research, we proposed that a measure of maternal ambivalence should include the following three aspects, namely Doubts about willing to be a mother or about being a good mother [2, 12, 21], Rejection (as opposed to Conviction) about being a mother [2, 12, 21, 22], and the Coping strategy (suppression/expression) used to deal with ambivalence [14, 24]. Encouragingly, the factor analyses, both when conducted in an exploratory and in a confirmatory manner, replicated this three-factor solution and the items were distributed as anticipated. This result is important because it represents the first attempt to provide a broad and exhaustive evaluation of maternal ambivalence based on a robust theoretical definition of the construct at issue. Also importantly, we proposed a second-order factor, which we labelled total MAS score, which can be used as a combination of all ambivalence factors within a single score.

The Doubts factor is the one that better represents the original definition of ambivalence. It refers to the co-existence of positive and negative evaluations and feelings regarding motherhood. A clear example of this is item 10: “When I think about motherhood, I have mixed positive and negative feelings.“ Not surprisingly, the Doubts dimension presented the strongest correlations with the measures used to evaluate the construct validity evidence of the MAS, namely anxiety, depression, and life satisfaction. These findings should be taken with caution due to the cross-sectional and non-experimental nature of the data, but one possible explanation for the results is that the presence of doubts about motherhood represents a source of discomfort in the mother, which might lead to anxiety and depression, especially if Doubts are maintained. Having doubts about maternity has been sometimes argued to be a natural and non-pathological process [12]. However, if unaddressed, research has also shown that doubts may cause feelings of uncertainty or inadequacy, fear of rejection, and other detrimental mental outcomes in the mother [12–14]. In line with these latter ideas, the present study results and past research [2] support the idea that identifying and reducing sources of doubt in women who experience recurrent and intense doubts about maternity during the perinatal period would be recommendable.

The Rejection of motherhood factor (as opposed to Conviction) refers to the meaning attributed to motherhood at that specific moment during a woman’s life, as well as her ideas about the relationship between maternity and her life purpose and identity. An example of this is item 4: “Being a mother at this time means moving forward and evolving in my life.“ Attributing a positive meaning to maternity and being confident about one’s will and ability to be a mother has been argued to positively impact well-being in the mother [2, 22]. Thus, as in the case of doubts, allocating professional support (e.g., midwifes, nurses, psychologists, or physicians) to increase perceived self-efficacy about maternity and to help mothers experience maternity as a more favourable period would be a sensible idea according to past research and the present study results. For example, a study revealed that perinatal women would like to receive information about the physical and psychological changes expected during pregnancy and after birth and development of the baby and they were generally open to receiving this information in an online format, which makes dissemination easier and cheaper [50].

Suppression is the third and final dimension in the MAS. In the maternal area, coping efforts have been generally conceptualized as a dichotomy between approach (i.e., seeking support and information when planning and preparing for maternity) and avoidance, such as attempts not to confront the challenges associated with preparing for maternity [14]. Approach in the MAS would be represented by items like “If I had doubts about motherhood, I would share them openly with a friend”, while avoidance would be represented by item “If I had any doubts about motherhood, I would probably keep them to myself.” Thus, Suppression in the MAS would evaluate the tendency to keep to oneself or to share ambivalent attitudes and feelings with people from the close circle (i.e., family, romantic partner, and close friends) as a strategy to deal with maternal ambivalence.

Expressing one’s emotions requires being aware of one’s internal states (e.g., maternal ambivalence) and allowing oneself to openly express such experience, while suppression implies less openness to one’s emotional states or to the consequences of sharing such emotions [51]. Not surprisingly, seeking emotional support is an adaptive emotion regulation strategy that minimizes the effects of stress and promotes well-being, as opposed to more inhibited coping styles that tend to lead to increased intrapersonal and interpersonal costs, such as depression, life dissatisfaction, and distancing by others [52, 53]. These detrimental interpersonal consequences of emotion suppression might be particularly relevant in maternity, because this might be a particularly challenging period which might be ameliorated if social support is present [54]. Our results support this idea that expression, in the case of ambivalence, would be preferable to suppression for the well-being (i.e., anxiety, depression, and life satisfaction) of mothers. Efforts should be made to encourage emotion expression, both by training mothers and their social acquaintances, but a model of flexibility in coping appears to be key [55]. In the case of mothers, this would refer to the ability to express or suppress one’s ambivalence depending on the situation. For example, it might be adequate to express ambivalence in an intimate situation with a romantic partner or a close friend (i.e., as evaluated in the MAS), where contingencies and potential misunderstandings might be more easily detected and dealt with, while suppression might be preferable in the presence of larger groups or in front of individuals with whom intimacy levels are low.

In addition to the findings in relation to the factor structure and construct validity of the MAS, another interesting finding was that some dimensions of ambivalence were different when comparing pregnant women and recent mothers (< less two years since delivery). It has been suggested that pregnant women are more likely to experience frustration compared to postpartum women because many of the experiences they undergo during pregnancy cannot be altered, such as the time until delivery, and also do not have the reinforcement of interacting with the baby-born [14]. This led us think that ambivalence would be higher during pregnancy. The results in this regard, however, were mixed. While the differences between both samples were not significant in the case of doubts, pregnant women were more likely to express their ambivalence, but less likely to be convinced about maternity than their counterparts. One first novel conclusion is that doubts about maternity might appear both during the gestation period and in the postpartum (i.e., two years after birth). This suggests that the postpartum experience would not be an element that solves doubts by itself, so the transition to motherhood might be a progressive and personal process for every woman that depends on a wider set of variables other than giving birth [2].

Regarding Rejection of motherhood, women who were already mothers were more confident and attributed a more positive meaning to motherhood than pregnant women. One possibility that explains this difference could lie in the bond between the mother and the baby. For example, it is possible that, once the baby is born and is “physical present and available” for the mother to interact with and receive positive reinforcements from (i.e., a smile or a funny face or noise), it becomes easier to attribute a meaning of personal growth to the experience and to be more enthusiastic about the experience (as in item “Being a mother is something that thrills me”). Another possibility is that the uncertainty during pregnancy about the development of childbirth, the baby’s health status, and one’s own experience of motherhood is positively influenced by delivery, thus positively impacting the Conviction dimension of ambivalence.

Like in Rejection, the expression of Doubts also differed across the two samples included in the study. This time, however, pregnant women showed a more encouraging outcome, particularly they were more likely to share their Doubts with others (i.e., relatives, romantic partner, and friends), while mother were keener to keep their doubts about motherhood to themselves. It is possible that women who are already mothers feel more social pressure to fulfil the tasks associated with motherhood and to show appreciation once the baby “is physically present”, so they might feel that they should suppress their doubts about motherhood, perhaps because they fear being judged and feel that it is their responsibility to know how solve the problems associated with motherhood [2]. Whatever the case, all these findings support the idea that programs aimed at targeting maternal ambivalence should be population-specific, in the sense that special emphasis should be made to enhance conviction and confidence about maternity during pregnancy, while emotional expression of ambivalence should be particularly encouraged during the postpartum to allow a healthier experience of maternity.

In the present study, the associations between maternal ambivalence and well-being have been interpreted as suggesting that ambivalence might be associated and probably lead to impaired emotional states and greater life dissatisfaction. Indeed, research has shown that maternal ambivalence is an unpleasant experience associated with mental distress [10, 14]. The debate on whether ambivalence is associated with negative affectivity or if ambivalence should be considered a strength [10] suggests that the former is more likely to represent the experience of ambivalence in pregnant women and mothers. Particularly, the presence of Doubts, Rejection, and the Suppression of ambivalence might boost or maintain the experience of unpleasant emotions such as anxiety and depressive symptoms. It is possible, however, that ambivalence appears or is enhanced by previous negative emotional states [2, 14]. Longitudinal studies are required to clarify this.

While this study has several strengths, including the solid theoretical background in scale development, the sample size, and the inclusion of women in two separate stages in the perinatal period (i.e., pregnant women and mothers of children under two years of age), the study also has some limitations. For example, as noted earlier during the text, the cross-sectional nature of the study prevents us from concluding whether the associations found are indicative of ambivalence leading to worse mood, worse mood leading to ambivalence, or both (as in a vicious cycle). Another limitation lies in the educational level and job status of the sample, which might be a consequence of the recruitment process used (i.e., online recruitment) and therefore impact the generalizability of the findings. In particular, 70% of the sample had university studies and 56% of them were active workers, so the results might not be representative of the general population of pregnant women and recent mothers in Spain. While the trend in the educational level in Spain show that women outnumber men in university studies [56], 70% is still not likely to be representative of the general population. As suggested by Koletzko and collaborators [14], who conducted a study with women who were mothers and who had professional careers prior to motherhood, it is possible that the degree of ambivalence is higher in people for whom motherhood consumes a large amount of temporary and economic resources that were previously destined to another area of ​​their lives that brought them satisfaction (i.e., active workers). The extent to which this is true would require comparing the findings of the present study with a large sample of individuals recruited from other sources (e.g., hospitals) and women who have less access to the Internet or have a basic level of digital literacy. Despite this, the large sample recruited represents a considerable effort to obtain a robust measure of maternal ambivalence and the reduced number of exclusion criteria to participate into the study should benefit the external validity of the results. Another source of potential bias other than the selection of participants was the selection of content in the development of the MAS. Even though this was minimized by consulting a multidisciplinary panel of five experts in the field, bias in the content validity of the questionnaire cannot be ruled out. As a final remark, it is important to note that, while maternal ambivalence might be particularly relevant during pregnancy and early after birth, ambivalence might also be important for women who are deciding whether they want to become mothers, for those who are actively seeking to become pregnant, for more experienced mothers, or for surrogate mothers [12, 57], to name some examples. While including these populations was out of the scope of the present investigation, an analysis of differences in ambivalence across these groups would also be of interest for future research.

## Conclusion

Studying the experiences that could lead to psychological problems that appear during pregnancy and maternity and that interfere with the mental health of women and their quality of life is of great importance to mobilize the care resources necessary to prevent and intervene in the mental health of women as soon as possible. For this, it is necessary to develop assessment instruments that allow to collect information about the presence of important underlying psychological processes, such as ambivalence regarding motherhood, and the degree of intensity and interference of them on well-being. The MAS is an important step in this direction. According to our results, the MAS might be a useful instrument to identify the presence of doubts during pregnancy and maternity, the conviction with the maternal role, and the coping strategies used to deal with ambivalence (i.e., expression/suppression). This information might be of great interest for clinical practice and the development of programs to promote well-being during pregnancy and after delivery, as well as for the prevention of psychological problems. In particular, this scale might be relevant for social workers, psychologists specialized in perinatal mental health, midwives, nurses, obstetricians, and gynaecologists. Far from pathologizing the experience of ambivalence, this study aims to provide a measure that allows professionals to detect when doubts are overwhelmingly present, conviction is very low, and the coping strategies used to deal with ambivalence are inadequate (i.e., excessive use of suppression). In sum, having a theoretically and psychometrically robust measure of ambivalence, like the MAS, allows health professionals to rapidly obtain this information, which is essential to normalize symptoms, develop programs and mobilize useful resources that aim to promote mental health in perinatal women.

## Supplementary Information

**Additional file 1.**

## Data Availability

The dataset used for the analyses can be found at: https://data.mendeley.com/datasets/rp26ck8hjy/1.

## Abbreviations

MAS: Maternal Ambivalence Scale
ODSIS: Overall Depression Severity Index Scale
OASIS: Overall Anxiety Severity Index Scale
SWLS: Satisfaction with Life Scale
CFA: Confirmatory Factor Analysis
RMSEA: Root Mean Square Error of Approximation
CFI: Comparative Fit Index
TLI: Tucker-Lewis Index
