# Supplementary material for: Development and psychometric properties of the maternal ambivalence scale in spanish women
Source: BMC Pregnancy Childbirth. 2022 Aug 6;22:625. doi: 10.1186/s12884-022-04956-w (PMC9356446; doi:10.1186/s12884-022-04956-w)
Supplement: Supplementary file 1 — Additional file 1. [file 12884_2022_4956_MOESM1_ESM.docx]

**Maternal Ambivalence Scale (English version)**

The following questions ask about thoughts you may be experiencing regarding current or future motherhood. Please indicate your level of agreement with each statement:

|  | Completely disagree | Somewhat disagree | Agree | Completely agree |
| --- | --- | --- | --- | --- |
| 1. Being a mother is something that thrills me |  |  |  |  |
| 1. I often doubt whether I really want to be a mother |  |  |  |  |
| 1. When I imagine or see myself interacting with my baby, I feel overwhelmed and insecure |  |  |  |  |
| 1. Being a mother right now means moving forward and evolving in my life |  |  |  |  |
| 1. I often find myself regretting being a mother |  |  |  |  |
| 1. Being a mother is something I want without a doubt |  |  |  |  |
| 1. I sometimes feel a great rejection or fear about motherhood |  |  |  |  |
| 1. It has always been clear to me that I want to be a mother |  |  |  |  |
| 1. I often want or have wanted to change my mind of the decision to become a mother |  |  |  |  |
| 1. When I think about motherhood, I have mixed positive and negative feelings |  |  |  |  |
| 1. If I had doubts about motherhood, I would share them openly with my family |  |  |  |  |
| 1. If I had doubts about motherhood, I would share them openly with a friend |  |  |  |  |
| 1. If I had doubts about motherhood, I would share them openly with my partner (if you don't have a partner, think about what you would do if you had a partner) |  |  |  |  |
| 1. If I had any doubts about motherhood, I would probably keep them to myself |  |  |  |  |

Doubts = items 2 + 3 + 5 + 7 + 9 + 10

Rejection = items 1+ 4 + 6 + 8

Suppression = items 11 + 12 +13 +14

**Escala de Ambivalencia Materna (Spanish version)**

Estas preguntas hacen referencia a pensamientos que puedes estar experimentando respecto a la maternidad, sea actual o futura. Indica tu nivel de acuerdo con cada afirmación:

|  | Totalmente en desacuerdo | En desacuerdo | De acuerdo | Totalmente de acuerdo |
| --- | --- | --- | --- | --- |
| 1. Ser madre es algo que me entusiasma |  |  |  |  |
| 1. A menudo dudo de si realmente quiero ser madre |  |  |  |  |
| 1. Cuando me imagino o me veo interaccionando con mi bebé me siento desbordada e insegura |  |  |  |  |
| 1. Ser madre en este momento significa avanzar y evolucionar en mi vida |  |  |  |  |
| 1. A menudo me veo arrepintiéndome del hecho de ser madre |  |  |  |  |
| 1. Ser madre es algo que quiero sin ninguna duda |  |  |  |  |
| 1. A veces me asalta un gran rechazo o miedo respecto a la maternidad |  |  |  |  |
| 1. Siempre he tenido claro que quería ser madre |  |  |  |  |
| 1. A menudo quiero o he querido echarme atrás en la decisión de ser madre |  |  |  |  |
| 1. Cuando pienso en la maternidad, tengo sentimientos positivos y negativos encontrados |  |  |  |  |
| 1. Si tuviera dudas respecto a la maternidad, las compartiría abiertamente con mi familia |  |  |  |  |
| 1. Si tuviera dudas respecto a la maternidad, las compartiría abiertamente con algún/a amigo/a |  |  |  |  |
| 1. Si tuviera dudas respecto a la maternidad, las compartiría abiertamente con mi pareja (si no tienes pareja, piensa en lo que harías si tuvieras pareja) |  |  |  |  |
| 1. Si tuviera dudas respecto a la maternidad, seguramente me las guardaría para mí misma |  |  |  |  |

Dudas = items 2 + 3 + 5 + 7 + 9 + 10

Rechazo = items 1+ 4 + 6 + 8

Supresión = items 11 + 12 +13 +14
